# Psychometric properties of the medical outcomes study: social support survey among methadone maintenance patients in Ho Chi Minh City, Vietnam: a validation study

**DOI:** 10.1186/s13011-018-0147-4

**Published:** 2018-02-14

**Authors:** Long Quynh Khuong, Tuong-Vi Thi Vu, Van-Anh Ngoc Huynh, Truc Thanh Thai

**Affiliations:** 1 Faculty of Public Health, Ho Chi Minh City University of Medicine and Pharmacy, 159 Hung Phu Street, Ward 8, District 8, Ho Chi Minh City, Vietnam; 2 South Vietnam HIV- Addiction Technology Transfer Center, Ho Chi Minh City University of Medicine and Pharmacy, 15th Floor, Central Building, 217 Hong Bang, Ward 11, District 5, Ho Chi Minh City, Vietnam; 3 Training and Scientific Research, University Medical Center, 215 Hong Bang Street, Ward 11, District 5, Ho Chi Minh City, Vietnam

**Keywords:** MOS-SSS, Social support, Methadone maintenance treatment, Psychometric property, Vietnamese

## Abstract

**Background:**

Social support plays a crucial role in the treatment and recovery process of patients engaging in methadone maintenance treatment (MMT). However, there is a paucity of research about social support among MMT patients, possibly due to a lack of appropriate measuring tools. This study aimed to evaluate the psychometric properties of the Vietnamese version of the Medical Outcomes Study: Social Support Survey (MOS-SSS) among MMT patients.

**Methods:**

A cross-sectional survey of 300 patients was conducted in a methadone clinic in Ho Chi Minh City, Vietnam. MMT patients who agreed to participate in the study completed a face-to-face interview in a private room. The MOS-SSS was translated into Vietnamese using standard forward-backward process. Internal consistency was measured by Cronbach’s alpha. The intra-class correlation coefficient was used to determine the test-retest reliability of the MOS-SSS in 75 participants two weeks after the first survey. Concurrent validity of the MOS-SSS was evaluated by correlations with the Multidimensional Scale of Perceived Social Support (MSPSS) and the Perceived Stigma of Addiction Scale (PSAS). Construct validity was investigated by confirmatory factor analysis.

**Results:**

The MOS-SSS had good internal consistency with Cronbach’s alpha from 0.95 to 0.97 for the four subscales and 0.97 for the overall scale. The two-week test-retest reliability was at moderate level with intra-class correlation coefficients of 0.61–0.73 for the four subscales and 0.76 for the overall scale. Strong significant correlations between the MOS-SSS and the MSPSS (*r* = 0.77; *p* < 0.001) and the PSAS (*r* = − 0.76; *p* < 0.001) indicated good concurrent validity. Construct validity of the MOS-SSS was established since a final four-factor model fitted the data well with Comparative Fit Index (0.97), Tucker-Lewis Index (0.97), Standardized Root Mean Square Residual (0.03) and Root Mean Square Error of Approximation (0.068; 90% CI = 0.059–0.077).

**Conclusions:**

The MOS-SSS is a reliable and valid tool for measuring social support in Vietnamese MMT patients. Further studies among methadone patients at different stages of their treatment and among those from different areas of Vietnam are needed.

**Electronic supplementary material:**

The online version of this article (10.1186/s13011-018-0147-4) contains supplementary material, which is available to authorized users.

## Background

Illicit drug use has long been recognized as a major global public health issue. It causes a lot of psychological and physical health consequences, such as depression, anxiety, psychosocial dysfunction, fatal and nonfatal overdose, and increases the risk of HIV transmission and other blood-borne diseases [1, 2]. There were 250 million drug users worldwide in 2015, with 29.5 million suffered from substance use disorders, approximately 1.6 million living with HIV and more than 6.1 million infected with hepatitis C [3]. Vietnam is a developing country located in the South-East Asia, in an area of 331,210 km^2^ and has the population of 92.7 million people [4]. In Vietnam, people who inject drugs have been found to be the primary drivers of the HIV epidemic, accounting for 34% of new HIV cases [5] and approximately 65% of all people living with HIV [6].

To address this issue, the methadone treatment has been considered as a priority in Vietnam due to its high degree of effectiveness not only in reducing the frequency of illicit drug use, HIV-related risk behaviors and illegal activities, but also in improving the general health and quality of life among drug users [7–10]. Since its first introduction in 2008, there are 280 methadone clinics to date, treating for 51,318 patients in the country, 95% were male [11]. However, since MMT involves long-term medication, patients are likely to have the risk of suffering withdrawal symptoms, drug relapse and drop out from MMT [12–14].

A large body of literature has demonstrated that social support is a significant predictor of success in methadone treatment and in the recovery process of drug users [15–17]. Data on social support is necessary to optimize the effectiveness of MMT. However, there is a lack of appropriate tools to measure this important component among Vietnamese patients undergoing MMT.

The Medical Outcome Study: Social Support Survey (MOS-SSS) [18] is one of the most widely used instruments. It is a brief, multi-dimensional scale developed to assess the functional aspects of perceived social support. The instrument is composed of 19 main items to measure four aspects of social support, including tangible support, emotional-informational support, positive social interactions and affectionate support. One additional item assesses the structural dimension of social support (i.e. the number of close relatives and friends) [18]. The high level of reliability and validity of the original MOS-SSS was demonstrated in a sample of 2987 chronic patients [18]. The MOS-SSS has been translated and adapted to different languages including Chinese [19, 20], Malay [21], French [22], Portuguese [23], Italian [24]. Since the questionnaires may be affected by the context in which they are used and differences in ethnicity and culture are likely to influence the way people understand and respond to the questionnaires [25], validation of the MOS-SSS is a crucial need.

The MOS-SSS has not been validated in Vietnam. The lack of such validated scale may result in the limited understanding of the social support levels among patients in MMT programs. This study was conducted to investigate the psychometric properties of the Vietnamese version of the MOS-SSS, including internal consistency, test-retest reliability, construct validity and concurrent validity among patients undergoing MMT in Vietnam.

## Methods

### Setting and participants

From May to July 2017, a cross-sectional study was conducted in Ho Chi Minh City which has the population of about 8.1 million people [4] and is the epicenter of drug use in Vietnam. There were approximately 22 thousand registered drug users and 4668 patients enrolled in the MMT program at 22 MMT clinics in the city [11]. The District 6 methadone clinic, one of the first MMT clinics in Vietnam, was selected for this study for its large number of patients with diversity in the duration of treatment. Vietnamese patients aged 18+ and currently on MMT were recruited using the simple random sampling technique. Among 318 patients invited to participate in the study, 300 patients (94%) agreed and completed the questionnaire. According to the empirical rule of thumbs that at least 3 to 10 patients per item are needed [26], and that a sample size of 300 is considered adequate for factor analysis [26, 27]. To evaluate test–retest reliability, the sample size was calculated to estimate the intra-class correlation coefficient (ICC), using the formula by Bonett, D.G [28]. With expected ICC of 0.78 [18], type 1 error rate of 5%, 95% confidence interval width of 0.2, at least 60 patients were needed. Two weeks after the first survey, 75 patients were asked to complete the same questionnaire.

### Procedures and measurements

Patients who agreed to participate in this study were invited to a private room to have a face to face interview in 20–25 min with the first author. Those who did not want to participate continued their usual treatment at the clinic.

We followed forward and backward translation process for translating the MOS-SSS [29]. A bilingual English school teacher translated the English version into Vietnamese. Another bilingual English school teacher who had no knowledge of the wording from the original English version conducted backward translation. The two translations were compared item by item and revised upon agreement among the authors and the two bilingual teachers. In Vietnamese culture, people do not usually hug to express their feelings, thus item 10 “*Someone who hugs you*” was replaced by “*Someone who gives you comforting gestures (such as hugging, holding hands)*” to ensure that the tool correctly assesses the affectionate support of respondents (see Additional file 1). A pilot study was conducted with 10 patients to check the wording [30]; these 10 patients were not included in the main survey. No changes were made during the pilot study.

#### Background information

The questionnaire included items about age, sex, marital status, religious affiliation, highest level of education completed, employment status and age of first drug use. The duration of MMT was extracted from medical records.

#### Medical outcome study – Social support survey (MOS-SSS)

The MOS-SSS consisted of 19 items (item 2 to item 20) measuring the functional aspects of perceived social support and one additional item (item 1) assessing the number of close relatives and friends (item 1 was not included in data analysis). The MOS-SSS measures four domains of social support including tangible support, emotional-informational support, positive social interactions and affectionate support [18]. Participants rated the MOS-SSS items using a five-point Likert rating scale ranging from (1) *none of the time* to (5) *most of the time*. The mean scores of the overall scale and four subscales were then transformed to a 100-point scale using the formula: Transformed score = [(*observed score* − *minimum possible score*)/(*maximum possible score* − *minimum possible score*)] × 100 [31]. A higher score indicates higher level of social support that patients perceive [18].

#### Multidimensional scale for perceived social support (MSPSS)

The scale is composed of 12 items measuring social support an individual perceives from family, friends and significant other. A 7-point Likert rating scale from (1) *very strongly disagree* to (7) *very strongly agree* is used. The overall score is the mean score of all items and ranges from 1 to 7, a higher score represents higher level of perceived social support. The MSPSS has good internal consistency with Cronbach’s alpha from 0.85 to 0.91 and adequate test-retest reliability. Construct validity and concurrent validity have also been established [32].

#### Perceived stigma toward addiction scale (PSAS)

The PSAS is a measure of perceived stigma toward those with substance use problems. Participants rate the PSAS items using a 7-point Likert-type rating scale ranging from (1) *strongly disagree* to (7) *strongly agree*. The PSAS is composed of 8 items, in which 2 items were scaled in a negative direction (higher score denotes higher perceived stigma) and 6 items in a positive direction, these 6 positive item scores then were reversed. A higher score of PSAS denotes higher perceived stigma [33]. The internal consistency of the PSAS is acceptable with Cronbach’s alpha = 0.73. Construct validity and concurrent validity have been demonstrated [33].

### Data analysis

We used frequencies and percentages to describe categorical variables, means and standard deviations to describe quantitative variables. The internal consistency of the MOS-SSS was measured by Cronbach’s alpha and item to total correlation coefficients. The Cronbach’s alpha measures the extent to which the items consistently measure the same thing [34], with the value of ≥0.80 indicating good internal consistency [27]. The item to total correlation indicates whether the response of every item is consistent with the average behavior of the scale. These correlation coefficients are Pearson’s correlation coefficients which ranges from 0 to 1, with the higher value indicating the better consistency. The test-retest reliability was evaluated using ICC, with the value > 0.75 indicating good reliability, 0.50–0.75 moderate reliability and < 0.50 poor reliability [35].

The concurrent validity of the MOS-SSS was examined using Pearson’s correlation coefficients. Construct validity was evaluated using confirmatory factor analysis (CFA) based on the originally established four-factor model [18]. CFA is a type of structural equation modeling that examines the latent structure of a test instrument (i.e., the relationships between observed measures or items and latent variables or factors). A fundamental feature of CFA is its hypothesis-driven nature, that is suitable to use when the previous evidence and theory of the model structure existed [36]. The Chi-squared statistic was used to identify whether the model fitted the data well. However, the Chi-squared is normally inflated by the large sample size and thus rejects the model [36]. The other fit indices including Comparative Fit Index (CFI), Tucker-Lewis Index (TLI), Standardized Root Mean Square Residual (SRMR) and Root Mean Square Error of Approximation (RMSEA) were used for model goodness of fit assessment. The CFI and TLI compare the model with alternative models such as a null or independence model in which the input indicators’ covariances are fixed to zero [36, 37]. The values of CFI and TLI > 0.9 indicate a well-fitting model [36, 38]. The SRMR is defined as the average discrepancy between the correlations observed in the input matrix and the correlations predicted by the model, while RMSEA incorporates a penalty function for poor model parsimony. These two fit indices indicate a ‘badness of fit’ or a ‘lack of fit’, thus the smaller value, the closer the fit between the model and the data [36, 37]. The values of SRMR and RMSEA (90% CI) < 0.08 indicate a good fit [36, 38]. All analyses were conducted using R version 3.4.0, packages *psych*, *irr* and *lavaan*.

## Results

### Participants’ characteristics

The majority of patients were male (92%), the mean age was 37 years (SD = 5.5 years) with 46.7% married or living with partners. Most of the patients attained secondary school or above (79%), were employed (62.3%) and had a religious affiliation (55.3%). The mean age of the first drug use was 19.7 years (SD = 4.8 years). The mean duration of MMT was 4.7 years (SD = 2.8 years) (Table 1).

**Table 1:** Socio-demographic characteristics of participants (*n* = 300)

Characteristics	Frequency	Percentage
Sex		
Male	276	92.0
Female	24	8.0
Marital status		
Single	101	33.7
Married/live with partners	140	46.7
Divorced/Separated/Widowed	59	19.6
Highest level of education		
Illiterate	11	3.7
Primary school	52	17.3
Secondary school	142	47.3
High school	75	25.0
College or more	20	6.7
Occupation		
Unemployed	113	37.7
Part-time job	80	26.7
Full-time job	107	35.6
Have a religious affiliation		
Yes	166	55.3
No	134	44.7
	Mean (SD)	Median (Min - Max)
Age, years	37.0 (5.5)	36.0 (26–58)
Age of first drug use, years	19.7 (4.8)	19.0 (12–41)
Duration on MMT, years	4.7 (2.8)	4.8 (0.17–9)

### Internal consistency and test-retest reliability

Table 2 shows the item distribution, internal consistency and test-retest reliability of the Vietnamese version of the MOS-SSS. The MOS-SSS had good internal consistency with Cronbach’s alpha of 0.97 for the overall scale and from 0.95 to 0.97 for the four subscales. The item to total correlation coefficients ranged from 0.77 to 0.88. The MOS-SSS had high level of two-week test-retest reliability with ICC of 0.76 for the overall scale and from 0.61 to 0.73 for the four subscales (Table 2).

**Table 2:** Item distribution, internal consistency and test-retest reliability of the MOS-SSS

Item^b^	Score [*n* (%)]^a^					Mean (SD)	Item-total correlation	Cronbach alpha	ICC^d^
	1	2	3	4	5				
Tangible^c^						66.7 (27.9)		0.97	0.73
Item 2: Help if you confined to bed	8 (2.7)	33 (11.0)	59 (19.7)	108 (36.0)	92 (30.6)	3.8 (1.0)	0.79		0.62
Item 5: Take you to the doctor	17 (5.7)	47 (15.7)	68 (22.7)	94 (31.3)	74 (24.6)	3.5 (1.2)	0.83		0.65
Item 12: Prepare meals for you	16 (5.3)	36 (12.0)	57 (19.0)	97 (32.3)	94 (31.4)	3.7 (1.2)	0.83		0.66
Item 15: Help you with daily chores	20 (6.7)	45 (15.0)	65 (21.7)	77 (25.6)	93 (31.0)	3.6 (1.3)	0.83		0.66
Emotional-informational^c^						47.5 (24.3)		0.97	0.72
Item 3: Listen to you	24 (8.0)	56 (18.7)	112 (37.3)	82 (27.3)	26 (8.7)	3.1 (1.1)	0.88		0.72
Item 4: Give you good advice	37 (12.3)	48 (16.0)	117 (39.0)	81 (27.0)	17 (5.7)	3 (1.1)	0.85		0.67
Item 8: Give you information	23 (7.7)	70 (23.3)	111 (37.0)	78 (26.0)	18 (6.0)	3 (1.0)	0.85		0.67
Item 9: Someone to confide in	43 (14.3)	80 (26.7)	74 (24.7)	90 (30.0)	13 (4.3)	2.8 (1.1)	0.80		0.59
Item 13: Give advice you really want	43 (14.3)	72 (24.0)	111 (37.0)	59 (19.7)	15 (5.0)	2.8 (1.1)	0.85		0.52
Item 16: Share worries with you	37 (12.3)	68 (22.7)	106 (35.3)	72 (24.0)	17 (5.7)	2.9 (1.1)	0.85		0.65
Item 17: Turn to for suggestions	33 (11.0)	65 (21.7)	112 (37.3)	76 (25.3)	14 (4.7)	2.9 (1.1)	0.85		0.59
Item 19: Understand your problems	51 (17.0)	66 (22.0)	100 (33.3)	74 (24.7)	9 (3.0)	2.8 (1.1)	0.84		0.52
Positive social interaction^c^						43.1 (25.5)		0.97	0.61
Item 7: Have a good time with you	37 (12.3)	79 (26.3)	108 (36.0)	65 (21.7)	11 (3.7)	2.8 (1.0)	0.77		0.57
Item 11: Get together for relaxation	36 (12.0)	91 (30.3)	100 (33.3)	57 (19.0)	16 (5.4)	2.8 (1.1)	0.77		0.49
Item 14: Help you get your mind off things	41 (13.7)	86 (28.7)	107 (35.7)	52 (17.3)	14 (4.6)	2.7 (1.1)	0.81		0.55
Item 18: Do something enjoyable with you	52 (17.3)	82 (27.3)	99 (33.0)	51 (17.0)	16 (5.3)	2.7 (1.1)	0.80		0.60
Affectionate^c^						54.6 (27.4)		0.95	0.64
Item 6: Show you love and affection	22 (7.3)	42 (14.0)	96 (32.0)	99 (33.0)	41 (13.7)	3.3 (1.1)	0.79		0.62
Item 10: Give you comforting gestures	34 (11.3)	67 (22.3)	79 (26.4)	81 (27.0)	39 (13.0)	3.1 (1.2)	0.82		0.54
Item 20: Love and make you feel wanted	24 (8.0)	67 (22.3)	81 (27.0)	95 (31.7)	33 (11.0)	3.2 (1.1)	0.81		0.57
Overall^c^						53.0 (22.9)		0.97	0.76

^a^1 = None of the time; 2 = A little of the time; 3 = Some of the time; 4 = Most of the time; 5 = All of the time

^b^Item 1 assesses the structural support (the number of close relatives and friends), and was not included in the analysis

^c^Scores were transformed to 100-point scale

^d^All ICCs were significant (*p* < 0.001)

### Construct validity and concurrent validity

**Fig. 1:**
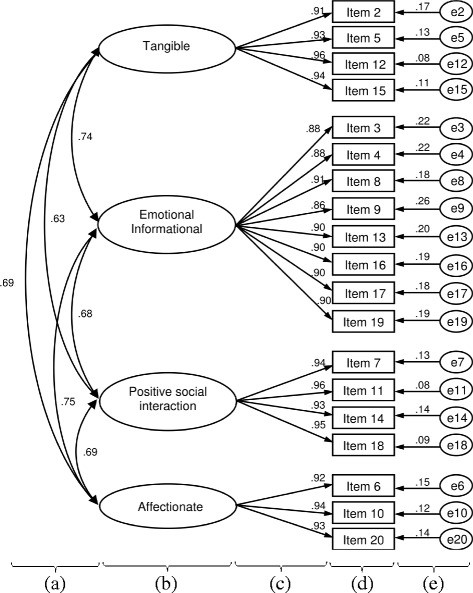
Confirmatory factor analysis for the four-factor model of the MOS-SSS. (**a**) Correlations between the factors (MOS-SSS subscales); (**b**) The MOS-SSS factors (MOS-SSS subscales); (**c**) Standardized factor loading; (**d**) The MOS-SSS item; (**e**) Error variance

The confirmatory factor analysis showed a significant difference between the final four-factor model and the expected model using the Chi-squared statistics (χ^2^ = 350.22, df = 146, *p* < 0.001). The CFI (0.97), TLI (0.97), SRMR (0.03), and RMSEA (0.068; 90% CI = 0.059–0.077) all revealed a good fit for the model specified (Fig 1). The four factors had strong correlations with one another, with correlation coefficients ranging from 0.63 to 0.75. Standardized factor loadings ranged from 0.86 to 0.96 and error variances were small (from 0.08 to 0.26), which indicate that the factors provide a good explanation of variation found in the items. Together, these data indicate that the MOS-SSS has adequate construct validity.

The correlation coefficients of the MOS-SSS with the MSPSS and the PSAS are shown in Table 3. The concurrent validity of the MOS-SSS was supported by significant positive correlations between the overall and subscales of the MOS-SSS and the MSPSS (*r* = 0.63–0.77, *p* < 0.001), and negative correlations with the PSAS (*r* = − 0.61– − 0.76, *p* < 0.001).

**Table 3:** The correlations between the MOS-SSS and the MSPSS and the PSAS

MOS-SSS	MSPSS	PSAS
Tangible	0.68^***^	−0.68^***^
Emotional-informational	0.72^***^	−0.69^***^
Positive social interaction	0.67^***^	−0.65^***^
Affectionate	0.63^***^	−0.61^***^
Overall scale	0.77^***^	−0.76^***^

^***^ *p* < 0.001

## Discussion

Although social support plays an important role in the treatment and recovery process of patients undergoing MMT, there has been no linguistically relevant instrument for measuring social support among this increasing population in Vietnam. The results of our study indicate that the MOS-SSS is a reliable and valid instrument for MMT patients in Ho Chi Minh City, Vietnam.

The MOS-SSS has good internal consistency and test-retest stability. The Cronbach’s alpha coefficients for the overall scale and four subscales were greater than 0.8, the threshold that is considered as a reasonable benchmark indicating good internal consistency. This result was similar to that reported for the original version, where Cronbach’s alpha ranged from 0.91 to 0.97 [18]. The value of the Cronbach’s alpha exceeding 0.95 might indicate the need for item redundancy [39]. However, given social support is a complex and multidimensional concept, the higher number of items is likely to correctly measure its various aspects [18, 40]. The high Cronbach’s alpha was also found in other validation studies, such as the Chinese version (α = 0.98) [19], Malay version (α = 0.96) [21], French version (α = 0.90–0.96) [22], Brazilian version (α = 0.95) [23]. Moreover, the high correlations between each item with the overall MOS-SSS provided evidence that all items were homogeneous in measuring the same construct and fulfilled the scaling assumption of internal consistency.

The stability of the MOS-SSS over a 2-week period was generally satisfactory with ICCs at moderate to good levels. This finding was consistent with that found in the original study where the stability of the scale was confirmed over a one year period [18]. Compared to other validation studies, the ICCs for the overall and four subscales of the MOS-SSS in our study were lower than those reported by Yu et al. (ICC = 0.84) [19], and Wang et al. (ICC = 0.74–0.89) [20] over a 2-week period. Since these two studies were conducted among inpatients with physical health problems, the condition of inpatients might facilitate the presence of higher stable level of support, such as the regular visits and informative support by physicians, or other support from relatives and friends. In contrast, drug users in Vietnam are still discriminated and stigmatized [13].

In terms of construct validity, although the Chi-squared test indicated that the model did not fit the data well, the other fit indices including CFI, TLI, SRMR and RMSEA revealed that the MOS-SSS was a good fit to a four-factor model. This four-factor solution was consistent with the original factor structure [18]. Other validation studies also reported the results that the Chi-squared test was unsatisfactory with the four-factor model but other fit indices showed a good fit [19, 20, 24]. Furthermore, the correlation among the four factors and high standardized factor loadings in the model were similar to those reported by Sherbourne & Stewart, where the correlation coefficients ranged from 0.69 to 0.82 and factor loadings ranged from 0.76 to 0.93 [18].

As expected, the MOS-SSS had good concurrent validity since it had positive correlation with MSPSS score (*r* = 0.77) and negative correlation with PSAS score (*r* = − 0.76). These findings were similar with the results reported by Yu et al. [19] where the correlation coefficients between the MOS-SSS subscale and MSPSS scale were high (*r* = 0.76–0.85).

Vietnam has shown intense efforts to reduce the number of drug users as well as the incidence of drug injection-related blood-borne diseases at both national and international levels, such as the plan to extend MMT service to 80,000 drug users [41]. Since social support highly affects the treatment success and recovery process of MMT patients [15–17], measurement of social support will yield important information to the relevant stakeholders in improving the quality of methadone treatment outcomes and ultimately respond to the epidemic of opioids abuse as well as its consequences. Researchers and health professional can use the Vietnamese version of MOS-SSS as screening tool for routine clinical care for methadone patients. This scale has been shown to be simple, cover the broad functional aspects of social support and have high level of reliability and validity. Such applications can help to fulfill the gaps in the paucity of information about social support and to improve quality of life in this vulnerable population in Vietnam.

The present findings should be interpreted in the context of a number of potential limitations. Since the validated scales for measuring social support are limited in literature, the MSPSS and the PSAS have not been validated in Vietnamese MMT patients and thus the concurrent validity found in this study might be potentially biased. Second, social support may be different and be specific to certain types of co-mordibility and health conditions such as HIV status and depression. Further studies investigating the psychometric properties of the MOS-SSS among MMT patients with different health conditions are needed. Third, although the characteristics of the MMT patients involved in this study were similar to previous studies in Vietnam and in Ho Chi Minh City in particular, including the high percentage of males of 90% - 95% [7, 9, 10, 42], all the patients in our study were from a methadone clinic in a large city and might not be generalizable to all MMT patients in other areas of Vietnam.

## Conclusions

Findings from our study demonstrated that the Vietnamese version of the MOS-SSS is a reliable and valid instrument in assessing the functional aspects of perceived social support for Vietnamese MMT patients. Further studies among methadone patients at different stages of their treatment and among those from different areas of Vietnam are needed.

## Additional file

Additional file 1: Vietnamese version of the Medical Outcome Study: Social Support Survey. (DOCX 18 kb)

## Abbreviations

CFI: Comparative fit index
HIV: Human immunodeficiency virus
ICC: Intra-class correlation coefficient
MMT: Methadone maintenance treatment
MOS-SSS: Medical Outcome Study – Social Support Survey
MSPSS: Multidimensional scale for perceived social support
PSAS: Perceived stigma toward addiction scale
RMSEA: Root mean square error of approximation
SRMR: Standardized root mean square residual
TLI: Tucker-lewis index
