# Supplementary material for: Psychometric properties of the medical outcomes study: social support survey among methadone maintenance patients in Ho Chi Minh City, Vietnam: a validation study
Source: Subst Abuse Treat Prev Policy. 2018 Feb 14;13:8. doi: 10.1186/s13011-018-0147-4 (PMC5813430; doi:10.1186/s13011-018-0147-4)
Supplement: Supplementary file 1 — Vietnamese version of the Medical Outcome Study: Social Support Survey. (DOCX 18 kb) [file 13011_2018_147_MOESM1_ESM.docx]

**Additional file 1:** Vietnamese version of the Medical Outcome Study: Social Support Survey

1. About how many close friends and close relatives do you have? *(people you feel at ease with and can talk to about what is on your mind)?*

1. Anh/chị có bao nhiêu bạn thân và người thân? *(những người mà anh/chị thấy dễ chịu khi tiếp xúc và có thể thoải mái tâm sự về những gì anh/chị* *đang suy nghĩ)*

| **No** | **Item** | **None of the time** (Không bao giờ) | **A little of the time** (Hiếm khi) | **Some of the time** (Thỉnh thoảng) | **Most of the time** (Thường xuyên) | **All of the time** (Luôn luôn) |
| --- | --- | --- | --- | --- | --- | --- |
| **Tangible support (Hỗ trợ hữu hình)** | | | | | | |
| 2 | Someone to help you if you were confined to bed  Có người nào đó sẽ giúp đỡ nếu anh/chị phải nằm một chỗ | 1 | 2 | 3 | 4 | 5 |
| 5 | Someone to take you to the doctor if you needed it  Có người nào đó sẽ đưa anh/chị đi khám bệnh nếu anh/chị cần | 1 | 2 | 3 | 4 | 5 |
| 12 | Someone to prepare your meals if you were unable to do it yourself  Có người nào đó nấu ăn/mua thức ăn cho anh/chị nếu anh/chị không tự làm được | 1 | 2 | 3 | 4 | 5 |
| 15 | Someone to help with daily chores if you were sick  Có người nào đó giúp anh/chị làm những việc hàng ngày nếu anh/chị bị ốm | 1 | 2 | 3 | 4 | 5 |
| **Emotional - informational support (Hỗ trợ cảm xúc – thông tin)** | | | | | | |
| 3 | Someone you can count on to listen to you when you need to talk  Có người nào đó sẽ lắng nghe khi anh/chị cần nói chuyện | 1 | 2 | 3 | 4 | 5 |
| 4 | Someone to give you good advice about a crisis  Có người nào đó sẽ cho lời khuyên hữu ích khi anh/chị gặp rắc rối | 1 | 2 | 3 | 4 | 5 |
| 8 | Someone to give you information to help you understand a situation  Có người nào đó cho anh/chị những thông tin giúp anh/chị hiểu rõ vấn đề | 1 | 2 | 3 | 4 | 5 |
| 9 | Someone to confide in or talk to about yourself or your problems  Có người nào đó mà anh/chị có thể tâm sự về bản thân hay những vấn đề rắc rối | 1 | 2 | 3 | 4 | 5 |
| 13 | Someone whose advice you really want  Có người nào đó mà anh/chị thật sự muốn họ cho lời khuyên | 1 | 2 | 3 | 4 | 5 |
| 16 | Someone to share your most private worries and fears with  Có người nào đó để anh/chị có thể chia sẻ những sự sợ hãi và lo lắng riêng tư | 1 | 2 | 3 | 4 | 5 |
| 17 | Someone to turn to for suggestions about how to deal with a personal problem  Có người nào đó mà anh/chị có thể hỏi ý kiến để giải quyết những rắc rối cá nhân | 1 | 2 | 3 | 4 | 5 |
| 19 | Someone who understands your problems  Có người nào đó hiểu rõ những vấn đề của anh/chị | 1 | 2 | 3 | 4 | 5 |
| **Affectionate support (Hỗ trợ tình cảm)** | | | | | | |
| 6 | Someone who shows you love and affection  Có người nào đó thể hiện tình thương và sự yêu mến với anh/chị | 1 | 2 | 3 | 4 | 5 |
| 10 | Someone who hugs you  Có người nào đó có những cử chỉ an ủi, vỗ về anh/chị *(ví dụ như ôm, nắm tay...)* | 1 | 2 | 3 | 4 | 5 |
| 20 | Someone to love and make you feel wanted  Có người nào đó để anh/chị yêu thương và khiến anh/chị cảm thấy mình được cần đến | 1 | 2 | 3 | 4 | 5 |
| **Positive social interaction (Tương tác xã hội tích cực)** | | | | | | |
| 7 | Someone to have a good time with  Có người nào đó cùng anh/chị có khoảng thời gian vui vẻ | 1 | 2 | 3 | 4 | 5 |
| 11 | Someone to get together with for relaxation  Có người nào đó cùng anh/chị thư giãn | 1 | 2 | 3 | 4 | 5 |
| 14 | Someone to do things with to help you get your mind off things  Có người nào đó cùng anh/chị làm những việc khiến anh/chị quên đi buồn phiền | 1 | 2 | 3 | 4 | 5 |
| 18 | Someone to do something enjoyable with  Có người nào đó cùng anh/chị làm những việc thú vị | 1 | 2 | 3 | 4 | 5 |
